# Hospital discharges-based search of acute flaccid paralysis cases 2007–2016 in Italy and comparison with the National Surveillance System for monitoring the risk of polio reintroduction

**DOI:** 10.1186/s12889-019-7617-0

**Published:** 2019-11-15

**Authors:** Paola Stefanelli, Stefania Bellino, Stefano Fiore, Stefano Fontana, Concetta Amato, Gabriele Buttinelli, the Regional Reference Centres of the National Surveillance System for Acute flaccid paralysis, Filippo Ansaldi, Sandro Binda, Laura Pellegrinelli, Guglielmo Bonaccorsi, Chiara Lorini, Silvio Brusaferro, Barbara Camilloni, Benita Capannolo, Cristiana Mancini, Valter Carraro, Paolo Castiglia, Antonella Arghittu, Marcello Mario D’Errico, Carlo De Stefano, Alfredo Focà, Cinzia Germinario, Angela Larocca, Giovanni M. Giammanco, Simona De Grazia, Guido Maria Grasso, Daniela Lombardi, Francesca Russo, Giuseppina Napoletano, Francesca Zanella, Silvia Spertini, Licia Veronesi, Paola Affanni, Maria Triassi, Francesca Pennino, Francesco Vairo

**Affiliations:** Department of Infectious Diseases, Italian National Institute of Health (Istituto Superiore di Sanità), Rome, Italy

**Keywords:** Acute flaccid paralysis, Hospital discharge records, National surveillance system, Polio

## Abstract

**Background:**

Acute flaccid paralysis (AFP) surveillance has been adopted globally as a key strategy for monitoring the progress of the polio eradication initiative. Hereby, to evaluate the completeness of the ascertainment of AFP cases in Italy, a hospital-discharges based search was carried out.

**Methods:**

AFP cases occurring between 2007 and 2016 among children under 15 years of age were searched in the Italian Hospital Discharge Records (HDR) database using specific ICD-9-CM diagnostic codes. AFP cases identified between 2015 and 2016 were then compared with those notified to the National Surveillance System (NSS).

**Results:**

Over a 10-year period, 4163 hospital discharges with diagnosis of AFP were reported in Italy. Among these, 956 (23.0%) were acute infective polyneuritis, 1803 (43.3%) myopathy, and 1408 (33.8%) encephalitis, myelitis and encephalomyelitis. During the study period, a decreasing trend was observed for all diagnoses and overall the annual incidence rate (IR) declined from 5.5 to 4.5 per 100,000 children. Comparing NSS with HDR data in 2015–2016, we found a remarkable underreporting, being AFP cases from NSS only 14% of those recorded in HDR. In particular, the acute infective polyneuritis cases reported to NSS accounted for 42.6% of those detected in HDR, while only 0.9% of myopathy cases and 13.1% of encephalitis/myelitis/encephalomyelitis cases have been notified to NSS. The highest AFP IRs per 100,000 children calculated on HDR data were identified in Liguria (17.4), Sicily (5.7), and Veneto (5.1) Regions; regarding the AFP notified to the NSS, 11 out of 21 Regions failed to reach the number of expected cases (based on 1/100,000 rate), and the highest discrepancies were observed in the Northern Regions. Overall, the national AFP rate was equal to 0.6, therefore did not reach the target value.

**Conclusions:**

AFP surveillance data are the final measure of a country’s progress towards polio eradication. The historical data obtained by the HDR have been useful to assess the completeness of the notification data and to identify the Regions with a low AFP ascertainment rate in order to improve the national surveillance system.

## Background

The World Health Organization (WHO) recommends countries to monitor cases of acute flaccid paralysis (AFP), which is the main clinical picture of a poliovirus infection, and the poliovirus (PV) circulation, to provide accurate data for the development of appropriate prevention and control strategies [1]. An important prerequisite for polio-free certification is that the National Surveillance System (NSS) successfully detects annually at least one case of non-polio associated AFP per 100,000 children under 15 years of age and no cases of wild polio occur for three consecutive years [1, 2]. The adequacy and quality of the AFP surveillance are assessed by the following key indicators recommended by the WHO: completeness of reporting, geographical and demographic representativeness of reporting sites, sensitivity of surveillance, completeness of case investigations and follow-up, laboratory performance [1]. Although low levels of PV transmission have been reported in recent years, the virus is still endemic in Nigeria, Afghanistan, and Pakistan. Until the PV transmission is not interrupted, all countries remain at risk of virus importation, through population movements. Indeed, polio eradication is hampered by the international spread of poliovirus through travellers. In response to the ongoing importations of poliovirus in polio-free countries, the WHO Director General on 5th May 2014 declared the international spread of wild poliovirus to be a public health emergency of international concern [3].

In Italy, AFP surveillance was established in 1994, according to the WHO guidelines, but started in January 1996 as a pilot study on four Italian Regions, and was finally extended to the whole nation in 1997. The Italian National Institute of Health (Istituto Superiore di Sanità – ISS) and the Ministry of Health (MoH) coordinate the national AFP surveillance system including 20 Regional Reference Centres (RRC).

Acute flaccid paralysis is defined as any case of new onset of hypotonic weakness in a child younger than 15 years of age and includes possible diseases due to paralytic poliomyelitis, Guillain-Barré Syndrome (GBS), transverse myelitis, polyradiculoneuritis, traumatic and neoplastic neuritis [4].

The present study aims to investigate the number of AFP cases reported from 2007 to 2016 in the Hospital Discharge Records (HDRs). These data were then compared with those obtained by the NSS in 2015 and 2016 for each Italian Region, in order to assess the completeness of reporting to the surveillance system.

## Methods

### Data sources

#### Hospital discharge records

All the hospitalizations made in public and private Italian hospitals are routinely collected in the HDR database, a systematic data collection on health care at national level, including both demographic and clinical information, such as primary and secondary diagnoses, primary and secondary diagnostic/therapeutic procedures, type of discharge, and hospital length of stay [5]. Diagnoses and procedures are coded using the “International Classification of Diseases, 9^th^ Revision, Clinical Modification” (ICD-9-CM).

We selected hospital discharges, among children aged less than 15 years, due to AFP based on the following ICD-9-CM codes as indicated in primary or secondary diagnoses [6]:

- ◦ 045.0X “Acute paralytic poliomyelitis specified as bulbar”
- ◦ 045.1X “Acute poliomyelitis with other paralysis”
- ◦ 357.0 “Acute infective polyneuritis” (approximates GBS)
- ◦ 359.9 “Myopathy, unspecified” (approximates Flaccid paralysis)
- ◦ 323.9 “Unspecified causes of encephalitis, myelitis, and encephalomyelitis” (approximates Acute transverse myelitis)

#### The Italian National Surveillance System for AFP

The active surveillance system was established at a national level in 1997, and it is coordinated by the ISS hosting the WHO collaborative centre for reference and research on polio. All AFP notifications included clinical and laboratory information and followed-up for residual paralysis at 60–90 days to ensure that the cases were not due to PV [4]. In particular, according to the WHO-recommended surveillance standard of poliomyelitis, the following criteria were considered:

- ◦ *clinical case definition*: any child under 15 years of age with AFP or any person of any age with paralytic illness if polio is suspected;
- ◦ *case classification*: suspected case (meets the clinical case definition) or confirmed case (presence of poliovirus in stool samples).

For all notified AFP to the RRC, stool samples were collected for laboratory confirmation. After 60 days from case notification, a follow-up questionnaire with more detailed information has to be sent to ISS and MoH to clarify the conclusive diagnosis. A first performance indicator is the number of AFP cases not due to polio per 100,000 children < 15 years of age per year, which is indicative of the sensitivity of the surveillance system (≥1/100,000). A second indicator is the proportion of AFP cases (≥80%) for which stool specimens in “good” conditions were collected [7].

According to the national regulation, ethics approval was not required as clinical isolates were collected, processed, and stored as part of routine clinical care by the hospital laboratories participating in the network. The surveillance activities are required by the Global Polio Eradication Initiative (GPEI) of the WHO in the “Polio Eradication & Endgame Strategic Plan 2013-2018”, and by the Italian Ministry of Health (ref. 0004114–08/02/2018-DGPRE-DGPRE-P).

Data used for our study was not publically available.

### Statistical analysis

All the HDRs between 1st of January 2005 and 31st of December 2016 were extracted from the MoH database. We considered the first hospital admission in case the same patient experienced more than one event, and then we excluded all hospitalization events occurring in the years 2005 and 2006, in order to exclude patients with sequelae from previous AFP discharges.

The number of hospitalizations, both overall and by specific diagnostic codes, and the AFP incidence rates were summarized by calendar year, gender, age groups, and geographical area (i.e., Northern, Central, and Southern Italy, based on the Italian National Institute of Statistics classification – ISTAT).

For the comparison between HDR and NSS data, we focused on the last 2 years of the considered period, 2015–2016, to study in-depth not only the differences between the two databases for each Italian Region, but also to highlight the discrepancies among Regions and how those vary in the latest data available year. Therefore, the absolute number and incidence rates (IR) of AFP coming from HDR data were calculated for each Region and compared with those reported by the NSS in the same period. Moreover, differences between the number of reported and expected cases notified to the NSS were calculated, to show the degree to which a Region was over or under the target value.

Statistical analysis was performed using the Stata software, version 13 (Stata Cooperation, College Station, Texas, USA).

## Results

We identified 4163 hospital events with a diagnosis compatible with AFP between 2007 and 2016 in Italy. Most cases were clinically diagnosed as unspecified myopathy (1803, 43.3%), followed by encephalitis/myelitis/encephalomyelitis (1408, 33.8%), and acute infective polyneuritis (956, 23.0%) (Table 1). A decreasing trend was observed for all diagnoses during the study period, and overall the annual AFP incidence rate (IR) declined from 5.5 to 4.5 per 100,000 children. In addition, the proportion of the three main diagnoses out of the total AFP cases for each calendar year remained quite stable over time. It is of note that 3 “Acute paralytic poliomyelitis specified as bulbar” cases (2 cases 045.00 unspecified type poliovirus, 1 case 045.02 poliovirus type I), and 1 “Acute poliomyelitis with other paralysis” case (045.10 unspecified type poliovirus) were incorrectly coded as ascertained after an investigation of ISS and MoH on medical records. A single vaccine-associated event, occurred in an Albanian subject before his arrival in Italy in 2014, was recorded as “Acute poliomyelitis with other paralysis, poliovirus type III”.

**Table 1:** Acute flaccid paralysis cases and incidence rates, Italy 2007–2016

ICD-9-CM diagnosis codes			045.0		045.1		357.0		359.9		323.9	
Year	AFP	IR	N	%	N	%	N	%	N	%	N	%
2007	457	5.5	0	0.0	0	0.0	117	25.5	171	37.3	169	36.9
2008	463	5.5	0	0.0	0	0.0	95	20.5	217	46.9	152	32.8
2009	473	5.6	0	0.0	0	0.0	101	21.3	196	41.4	176	37.1
2010	434	5.1	0	0.0	0	0.0	110	25.2	195	44.7	130	29.8
2011	443	5.2	0	0.0	0	0.0	113	25.5	190	42.9	140	31.6
2012	412	4.9	0	0.0	0	0.0	88	21.4	171	41.5	155	37.6
2013	378	4.5	0	0.0	0	0.0	84	22.2	174	46.0	120	31.7
2014	390	4.6	0	0.0	1	0.3	93	23.8	166	42.6	130	33.3
2015	339	4.0	0	0.0	0	0.0	62	18.3	162	47.8	116	34.2
2016	374	4.5	0	0.0	0	0.0	93	24.9	161	43.0	120	32.1
2007–2016	4163	5.0	0	0.0	1	0.0	956	23.0	1803	43.3	1408	33.8

Data were extracted from Hospital Discharge Records [5]

Italian population data were obtained from ISTAT database (www.demo.istat.it)

Five cases presented more than one diagnosis

ICD-9-CM diagnosis codes: 045.0 “Acute paralytic poliomyelitis specified as bulbar“;

045.1 “Acute poliomyelitis with other paralysis“; 357.0 “Acute infective polyneuritis”;

359.9 “Myopathy, unspecified”; 323.9 “Unspecified causes of encephalitis, myelitis, and encephalomyelitis”

357.0 code approximates Guillain-Barré Syndrome; 359.9 code approximates Flaccid paralysis; 323.9 code approximates Acute transverse myelitis

*AFP* acute flaccid paralysis, *IR* incidence rate × 100,000

Table 2 shows the main characteristics of the AFP cases among children aged 0–14 years reported in HDR database in the last 2 years of the study period, 2015–2016. Females were less frequently affected by AFP as compared to males [incidence rate ratio (IRR) 0.7/100,000], and fewer cases of AFP were recorded among children aged 5–9 and 10–14 years compared to those aged 0–4 years (IRR 0.6 and 0.5 per 100,000). In addition, small differences in the AFP incidence rates were found among the three geographic areas, as the Central and Southern Italy compared to Northern Italy (the most populated area, and with more hospital services in the paediatric setting) reported an IRR of 0.9 and 0.8, respectively. Similar patterns of AFP types were observed in all geographic areas. The most common cause of AFP cases was myopathy (IR 1.9/100,000), followed by encephalitis/myelitis/encephalomyelitis (IR 1.4/100,000) and acute infective polyneuritis (0.9/100,000).

**Table 2:** Demographic and clinical characteristics of Acute Flaccid Paralysis cases, Italy 2015–2016

AFP = 713	Number	Percent	IR	IRR	95% CI IRR
Gender					
Male	430	60.3	5.0	–	
Female	283	39.7	3.5	0.7	0.6–0.8
Class of age (years)					
0–4	322	45.2	6.2	–	
5–9	198	27.8	3.5	0.6	0.5–0.7
10–14	193	27.1	3.4	0.5	0.5–0.7
Nationality					
Italian	627	87.9	4.2	–	
Other	86	12.1	4.6	1.1	0.9–1.4
Geographic area					
North	357	50.1	4.7	–	
Centre	132	18.5	4.1	0.9	0.7–1.1
South	224	31.4	3.8	0.8	0.7–1.0
Clinical characteristics					
Acute infective polyneuritis (357.0)	155	21.7	0.9	–	
Myopathy, unspecified (359.9)	323	45.2	1.9	2.1	1.7–2.6
Unspecified causes of encephalitis, myelitis, and encephalomyelitis (323.9)	236	33.1	1.4	1.6	1.3–1.9

Data were extracted from Hospital Discharge Records [5]

Italian population data were obtained from ISTAT database (www.demo.istat.it)

357.0 code approximates Guillain-Barré Syndrome; 359.9 code approximates Flaccid paralysis; 323.9 code approximates Acute transverse myelitis

*AFP* acute flaccid paralysis, *IR* incidence rate × 100,000 population, *IRR* incidence rate ratio, *CI* confidence interval

**Fig. 1:**
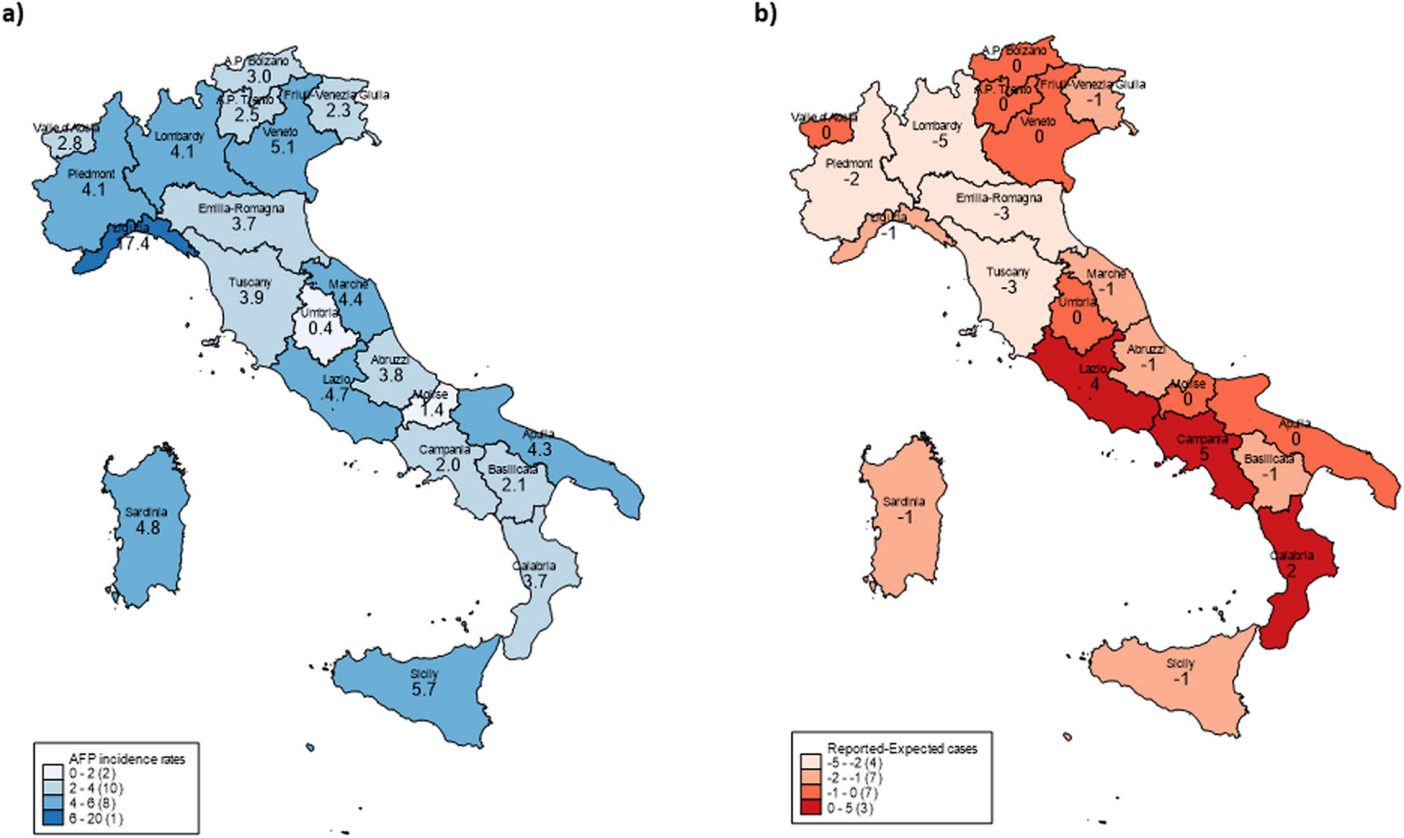
**a**-**b** Acute Flaccid Paralysis by Italian Regions, years 2015–2016. Incidence rates per 100,000 children, data from Hospital Discharge Records (**a**); Difference between the number of reported and expected cases, data from the National Surveillance System (**b**). The maps have been produced using Stata software, version 13 (Stata Cooperation, College Station, Texas, USA)

The AFP cases from HDR were compared to those reported to the NSS in 2015–2016, analysing data from each of the 20 Italian Regions (Trentino-Alto Adige Region is formed by Bolzano and Trento Autonomous Provinces) (Table 3). Twelve cases notified to the NSS were not included in the analysis, because they did not fit with the considered ICD-9-CM specific codes. Major AFP IR (per 100,000 children) coming from HDR data were detected in Liguria (17.4), Sicily (5.7) and Veneto (5.1), whereas the lowest values were recorded in Umbria (0.4) and Molise (1.4) (Fig. 1a). Of note, the highest percentages of hospitalization among non-residents were recorded in Liguria and Lazio Regions, nearly 64 and 40% respectively, indicating an interregional mobility. Regarding the AFP notified to the NSS, the completeness of reporting varied by Regions. Considering the difference between the number of reported and expected cases (based on 1/100,000 rate), 11 out of 21 Regions failed to achieve the target (values < 0), and the highest discrepancies were observed in the Northern Regions (Lombardy, Emilia-Romagna, Tuscany, and Piedmont) (Fig. 1b). Campania, Lazio and Calabria were over the expected value (difference > 0), while for 7 Regions no difference was found.

**Table 3:** Acute Flaccid Paralysis cases and incidence rates: comparison between Hospital Discharge Records and National Surveillance System, Italy 2015–2016

Regions	AFP 2015				357.0		359.9		323.9		AFP 2016				357.0		359.9		323.9	
	HDR	HDR IR	NSS	NSS IR	HDR	NSS	HDR	NSS	HDR	NSS	HDR	HDR IR	NSS	NSS IR	HDR	NSS	HDR	NSS	HDR	NSS
Piedmont	19	3.3	3	0.5	4	3	7	0	8	0	27	4.8	5	0.9	3	2	11	2	13	1
Valle d’Aosta	1	5.6	0	0.0	1	0	0	0	0	0	0	0.0	0	0.0	0	0	0	0	0	0
Lombardy	60	4.2	4	0.3	10	2	28	0	22	2	57	4.0	14	1.0	15	8	25	0	17	6
A.P. Bolzano	0	0.0	0	0.0	0	0	0	0	0	0	5	6.0	3	3.6	3	3	1	0	1	0
A.P. Trento	1	1.2	1	1.2	1	1	0	0	0	0	3	3.8	1	1.3	1	1	1	0	1	0
Veneto	33	4.8	3	0.4	4	3	16	0	13	0	37	5.4	10	1.5	10	9	12	0	15	1
Friuli-Venezia Giulia	3	1.9	0	0.0	1	0	2	0	1	0	4	2.6	0	0.0	2	0	2	0	0	0
Liguria	35	19.2	0	0.0	2	0	28	0	5	0	28	15.6	1	0.6	2	1	22	0	4	0
Emilia-Romagna	13	2.2	4	0.7	4	2	5	0	4	2	31	5.2	1	0.2	11	0	10	0	10	1
Tuscany	23	4.8	0	0.0	1	0	14	0	8	0	14	2.9	3	0.6	3	3	8	0	3	0
Umbria	0	0.0	1	0.9	0	0	0	0	0	1	1	0.9	0	0.0	0	0	1	0	0	0
Marche	8	3.9	1	0.5	3	0	3	0	2	1	10	5.0	1	0.5	3	0	6	0	1	1
Lazio	41	5.0	2	0.2	10	2	26	0	5	0	35	4.3	5	0.6	10	3	19	0	6	2
Abruzzi	6	3.5	2	1.2	1	1	1	0	4	1	7	4.1	0	0.0	1	0	2	0	4	0
Molise	1	2.7	0	0.0	0	0	0	0	1	0	0	0.0	0	0.0	0	0	0	0	0	0
Campania	16	1.8	7	0.8	6	4	7	0	3	3	20	2.2	0	0.0	5	0	10	0	5	0
Apulia	22	3.8	5	0.9	2	5	7	0	13	0	27	4.8	8	1.4	9	3	6	0	11	5
Basilicata	2	2.7	0	0.0	0	0	1	0	1	0	1	1.4	0	0.0	0	0	0	0	1	0
Calabria	7	2.6	1	0.4	3	1	2	0	2	0	13	4.8	0	0.0	4	0	3	0	6	0
Sicily	39	5.3	5	0.7	6	3	13	0	20	2	45	6.2	7	1.0	7	5	19	1	19	1
Sardinia	9	4.5	1	0.5	3	1	2	0	4	0	10	5.1	1	0.5	4	0	3	0	3	1
Italy	339	4.0	40	0.5	62	28	162	0	116	12	375	4.5	60	0.7	93	38	161	3	120	19

Data were extracted from Hospital Discharge Records; Italian population data were obtained from ISTAT database (www.demo.istat.it)

*AFP* acute flaccid paralysis, *HDR* Hospital Discharge Records [5], *NSS* National Surveillance System [4], *IR* Incidence rate. ICD-9-CM diagnosis codes: 357.0 “Acute infective polyneuritis”; 359.9 “Myopathy, unspecified”; 323.9 “Unspecified causes of encephalitis, myelitis, and encephalomyelitis”

Overall, the total number of AFP cases from NSS in Italy did not reach the expected value, which is nearly 83 cases considering an Italian population aged under 15 years of 8,300,000. In 2015–2016, AFP cases from NSS were 14% of those obtained by HDR data, indicating a high level of underreporting at a national level. Specifically, only 42.6% of acute infective polyneuritis cases, 13.1% of encephalitis/myelitis/encephalomyelitis cases, and 0.9% of myopathy cases reported in HDR were notified to the NSS.

## Discussion

The present study aimed to evaluate the AFP surveillance system over a 10-year period in Italy by comparing data obtained from HDR with those from NSS in the last 2 years by each Italian region. The comparison indicates that NSS data are affected by underreporting, since the AFP cases from NSS were only 14% of those recorded in HDR during the years 2015–2016. Specifically, the percentage of notified cases differs by clinical diagnosis being almost absent for unspecified myopathies (359.9 ICD-9-CM code), and very low for encephalitis/myelitis/encephalomyelitis with unspecified causes (323.9 ICD-9-CM code), while GBS cases (included in 357.0 ICD-9-CM code) have been notified. Even the underreporting exists, the NSS still plays a crucial role in identifying AFP cases that would remain undetected due to the disadvantage of HDRs, since they have the limitation that is not based on specific case definitions, and therefore not extremely accurate. This is demonstrated by coding errors for the discharge diagnoses of four not confirmed cases of poliomyelitis, lacking the requirement for a laboratory-based confirmation.

Although poliomyelitis has historically been responsible for most cases of flaccid paralysis infection-related, non-polio enteroviruses were well known to be associated with AFP/Acute Flaccid Myelitis (AFM) [8]. In particular, enterovirus D68 (EV-D68) has become increasingly recognized in the context of enhanced surveillance for polio-like diseases [9]. Large outbreaks of EV-D68 occurred in the United States, Canada, and Europe in 2014 and 2016, temporally and geographically associated with an increase in AFM [10]. During 2018, the United Kingdom described an increase in reports of AFP cases, with many cases diagnosed as the AFM occurred mainly among preschool children [11]. In Italy, data on EV-D68 circulation are not systematically collected in the context of AFP surveillance.

In Italy, the last case of indigenous poliomyelitis due to a wild poliovirus occurred in 1982, while in 1988 the last imported case of polio infection was reported. Polio vaccination became mandatory in 1966 for children within the first year of age; in 2002, the inactivated Salk polio vaccine (IPV) completely replaced the Sabin oral polio vaccine (OPV) for safety reasons and risk-benefit considerations. The current National Vaccination Plan for 2017–2019 includes a primary vaccination with three doses at 3, 5, and 11 months of age and booster doses at 6 and 12–18 years of age [12]. Currently, in the EU area, Bosnia and Herzegovina, Romania and Ukraine are considered countries with a high risk of polio outbreak following the importation of wild PV or emergence of circulating vaccine-derived PV due to suboptimal vaccination program performance and low immunity of the population [7]. Furthermore, several middle-income countries were considered at intermediate risk of polio transmission, as vaccination coverage appears to be declining and the quality of poliovirus surveillance is not optimal [13]. Moreover, in the three remaining endemic countries, Afghanistan, Pakistan and Nigeria, poliovirus transmission continued in 2018 [14].

In this scenario, maintaining a high vaccination coverage against polio minimizes the risk of spreading the disease in case of importation, and a highly sensitive surveillance system for AFP is essential to monitor polio-free status certification, and to respond to vaccine-derived polioviruses outbreaks, which could circulate for several years after ending the use of OPV.

As established by the WHO, at least 1 case of non-polio AFP should be detected annually per 100,000 population under 15 years of age, which is an adequate quality of the surveillance system [1]. However, since the beginning of the NSS, the average value of the non-polio AFP rate was 0.7 in Italy and reached the target level only in 2003. Of note, this value is in line with the average AFP detection rate (0.6/100,000) from 1998 to 2015 in Spain [15]. In this country, the quality of AFP surveillance has decreased over time, particularly after the switch from OPV to IPV, reflecting the loss of awareness about polio, when it is very unlikely that cases of paralysis produced by wild PV or associated with the vaccine-derived PV will occur. However, notifications and hospital admissions with a clinical diagnosis compatible with AFP have followed the same pattern, indicating that, despite the underreporting, the surveillance works relatively well. Overall, in regions certified as polio-free for many years (the Americas in 1994, Western Pacific Region in 2000, and WHO European Region in 2002 [2]), the sensitivity and quality of AFP reports decreased over time [16].

In our country, several possible reasons have been identified that contribute to the underreporting in AFP surveillance system: the long absence of polio in Italy with consequently loss of interest in preventive issues and maintainance of polio-free status; the lack of knowledge and awareness of clinicians to associate the surveillance with non-polio AFP; the presence of a confirmed alternative diagnosis. Specifically, some paediatricians could equate AFP only to acute poliomyelitis and not with a wide range of conditions that AFP can present, such as making a specific diagnosis of GBS rather than labelling a case as AFP. Indeed, the widespread eradication of poliomyelitis has led to GBS becoming the most frequent cause of acute and sub-acute flaccid paralysis. Furthermore, the scarce notification of AFP cases could be related to the physicians’ lack of knowledge of the surveillance criteria and of the importance of reporting and investigating all AFP cases, or finally could be due to management problems that are much more evident in some parts of Italy. Overall, several efforts could be made to enhance and strengthen the surveillance activities, by increasing awareness of optimal investigation and management of cases, as well as specific training to increase knowledge among clinicians, microbiologists, public health specialists and other key partners involved in notifiable disease reporting. In addition, laboratory testing for non-polio enteroviruses could be implemented both to monitor polio eradication and to establish sensitive surveillance to detect other enteroviruses that cause severe symptoms such as EV-D68.

## Conclusions

Our study shows that the Italian AFP surveillance system is affected by underreporting and that the completeness of reporting varies by Italian Regions. Therefore, it is important to closely monitor the activity of the various paediatric centres in the country to improve the quality of the surveillance system. Furthermore, the regular adoption of the cross-evaluation of notified data, also through the review of HDRs, could help to plan effective actions for the improvement of the NSS.

## Data Availability

Data supporting the results reported in the article include the Hospital discharge records and the National surveillance system database. HDR data are sent by hospitals to the Regional Health Authorities that transmit the database to the Ministry of Health. The database is available at the Istituto Superiore di Sanità as agreed with the “Collaboration agreement between the Ministry of Health and ISS for the use of Hospital discharge information flow to conduct studies in Public Health”. Regarding the AFP National Surveillance System database, all records are shared with the Italian Ministry of Health, as the competent body for the AFP surveillance in Italy. Data are available from the authors upon reasonable request and with permission of the Italian Ministry of Health.

## Abbreviations

AFP: Acute flaccid paralysis
GBS: Guillain-barré syndrome
HDR: Hospital discharge records
ICD-9-CM: International Classification of Diseases, 9th Revision, Clinical Modification
IPV: Inactivated salk polio vaccine
IR: Incidence rate
ISS: Istituto Superiore di Sanità
MoH: Ministry of Health
NSS: National Surveillance System
OPV: Oral polio vaccine
PV: Poliovirus
RRC: Regional Reference Centres
WHO: World Health Organization
